# Cardiovascular and renal burdens among patients with MAFLD and NAFLD in China

**DOI:** 10.3389/fendo.2022.968766

**Published:** 2022-09-02

**Authors:** Yuying Wang, Yuetian Yu, Haojie Zhang, Chi Chen, Heng Wan, Yi Chen, Fangzhen Xia, Shiyan Yu, Ningjian Wang, Lin Ye, Yingli Lu

**Affiliations:** Institute and Department of Endocrinology and Metabolism, Shanghai Ninth People’s Hospital, Shanghai JiaoTong University School of Medicine, Shanghai, China

**Keywords:** CVD, CKD, MAFLD, NAFLD, China

## Abstract

**Background/Purpose:**

Metabolic associated fatty liver disease (MAFLD) was proposed as a new definition to put emphasis on the metabolic aspects of nonalcoholic fatty liver disease (NAFLD). We aim to compare the cardiovascular and renal burden between MAFLD and NAFLD patients.

**Methods:**

12183 participants were enrolled in East China. The cardiovascular burden (Framingham risk score and previous cardiovascular diseases (CVD)) and renal burden (eGFR and chronic kidney disease (CKD)) were measured.

**Results:**

The risk of hypertension, dyslipidemia, diabetes, overweight/obesity, and central obesity of MAFLD patients were higher than those of NAFLD. Patients with MAFLD have a similar or higher beta coefficients in Framingham risk score [beta (95%CI): male 0.062 (0.055,0.069) vs 0.041 (0.033,0.048); female 0.014 (0.012,0.016) vs 0.012 (0.01,0.014)], and higher odds ratio in previous CVD [odds ratio (95%CI): male 1.50 (1.22,1.85) vs 1.35 (1.1,1.66); female 1.58 (1.33,1.87) vs 1.45 (1.22,1.72)], compared with those with NAFLD. However, compared with males with MAFLD, the odds ratio of CKD was higher in those with NAFLD [eGFR: -2.731 (-3.422, -2.041) vs-3.578 (-4.268, -2.887). CKD: 1.44 (1.05,1.96) vs 1.56 (1.14,2.12)]. In female, CKD was only marginally associated with NAFLD [0.8 (0.62,1.02), P=0.075], but not MAFLD [0.87 (0.68,1.11), P=0.268].

**Conclusions:**

Patients with MAFLD have a similar or higher risk of future and previous CVD compared with those with NAFLD, but the risk of CKD was higher in male with NAFLD.

## Introduction

Over the last decade, non-alcoholic fatty liver disease (NAFLD) has been the most common cause of chronic liver diseases all over the world, which affects about a quarter of the world’s adult population and poses a major health and economic burden to all societies (1). Given that obesity and type 2 diabetes mellitus (DM) are consistently considered as two most important risk factors for NAFLD (2), the emergence of new definition - metabolic associated fatty liver disease (MAFLD) instead of NAFLD put emphasis on the metabolic aspects of the disease (3).

Once considered NAFLD as a disease for the West, NAFLD and its complications now also pose a major health threat in China (4). Some researchers predict that China will have the highest growth in the prevalence of NAFLD all over the world, with about 300 million cases by 2030 (5). In addition, China is the youngest median age of NAFLD country in the world, which means that the impact of its late-stage complications will occur in the coming decades (5). However, there are few studies about the prevalence and characteristics of MAFLD, the new definition of NAFLD.

As for NAFLD is a multisystem disease, some evidences show that NAFLD is intimately associated with cardiovascular disease (CVD) that is the primary cause of premature death (6). According to the new definition of MAFLD, although it is reasonable to speculate that compared with NAFLD, MAFLD may be more related to the occurrence of CVD as well as other metabolism-related diseases, the actual situation needs to be confirmed (7, 8). Chronic kidney disease (CKD) is another worldwide health problem that results in high morbidity, mortality, and health care costs. As the presence of pathophysiological inter-relationships between the liver and the kidney is well established, the possible link between fatty liver and CKD has also attracted scientific interest (9) (10). The burdens of CKD in MAFLD is unclear and needs to be assessed (11).

A large ongoing investigation started in 2014, which is referred to as Survey on Prevalence in East China for Metabolic Diseases and Risk Factors (SPECT-China). We aim to understand the differences in the burdens of CVD and CKD in Chinese population with MAFLD and NAFLD.

## Methods

### Study population

**Figure 1:**
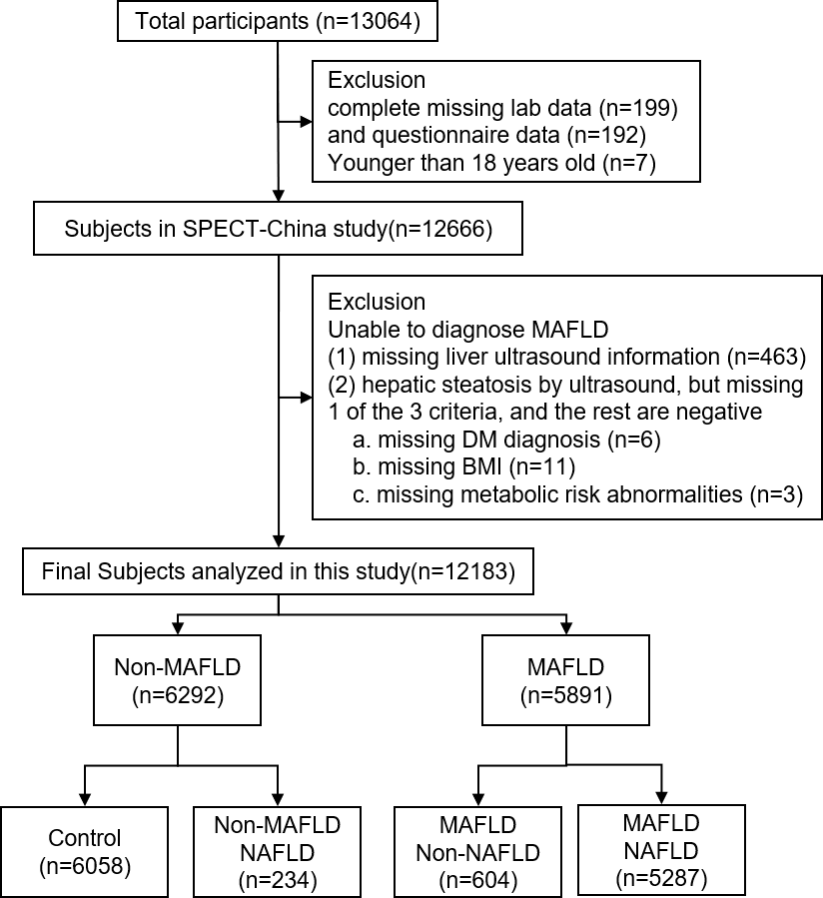
The inclusion and exclusion criteria of the study participants.

SPECT-China was a cross-sectional investigation of the prevalence of metabolic diseases and risk factors in East China (ChiCTR-ECS-14005052, www.chictr.org.cn). A stratified cluster sampling method was used to select a sample from the general population. In total, 13064 subjects were recruited. The exclusion criterion was the following: no blood sample submitted (n=199) and questionnaire data (n=192), younger than 18 years old (n=7), unable to diagnose MAFLD (missing liver ultrasound information (n=463), hepatic steatosis by ultrasound, but missing 1 of the 3 criteria, and the rest are negative: missing DM diagnosis (n=6), missing BMI (n=11), missing metabolic risk abnormalities (n=3)). Finally, 12183 participants were included in this study. These participants were then divided into four groups, the control group(n=6058), the Non-NAFLD MAFLD group(n=604), the Non-MAFLD NAFLD group(n=234) and the NAFLD MAFLD group(n=5287). The flowchart of participants was shown in **Figure 1**.

### Clinical, anthropometric, and laboratory measurements

The staff were trained according to a standard protocol as previously applied (12, 13). They used a questionnaire to collect information on participants’ demographic characteristics and lifestyle risk factors. Venous blood samples were drawn after an overnight fast of at least 8 hours. Hemoglobin A1c (HbA1c) was assessed by HPLC (MQ-2000PT, Medconn, Shanghai, China). Fasting plasma glucose (FPG), triglycerides (TG), total cholesterol (TC), high (HDL-C) and low-density lipoprotein (LDL-C), alanine transaminase (ALT) and aspartate transaminase (AST) were measured by a Beckman Coulter AU 680 analyzer and insulin by chemiluminescence (Abbott i2000 SR).

Abdominal ultrasonographic examination was performed in all subjects by two experienced ultrasonographers using an ultrasound device (MINDRAY M7). The diagnostic criteria for liver steatosis by ultrasonography included increased liver echogenicity, stronger echoes in the hepatic parenchyma than in the renal parenchyma, vessel blurring, and narrowing of the lumen of the hepatic veins (14).

### Definition of variables

MAFLD was defined by hepatic steatosis in adults, in addition to one of the following three criteria: general overweight/obesity, presence of T2DM, or evidence of metabolic dysregulation (3). High sensitive C reaction protein (hsCRP) was not examined in the study population, so it was not included in the evidence of metabolic dysregulation. NAFLD was defined as ultrasound evidence of fatty liver and the exclusion of secondary causes (having a history of excessive consumption (>20 g/d) of pure alcohol, self-reported viral hepatitis, using medications associated with secondary NAFLD (corticosteroids, amiodarone) (15).

Participants who met the diagnostic criteria of NAFLD and didn’t meet the diagnostic criteria of MAFLD were included in the Non-MAFLD NAFLD group and participants who met the criteria of MAFLD but not NAFLD were included in the Non-NAFLD MAFLD group. The group NAFLD-MAFLD included participants who met the diagnostic criteria of MAFLD and NAFLD at the same time, which means they had liver steatosis, had anyone of the following overweight/obesity, presence of T2DM, or metabolic dysregulation and without secondary causes of fatty liver. People who didn’t met the criteria of both NAFLD and MAFLD were considered as the control group.

For individuals 30 to 74 years old and without CVD at the examination, the mean 10-year risk of cardiovascular events was calculated by the modified Framingham risk score (16). Previous CVD included self-reported myocardial infarction and stroke. Estimated glomerular filtration rate (eGFR) was calculated by the Chronic Kidney Disease Epidemiology Collaboration (CKD-EPI) equation for “Asian origin” (17). CKD was defined as eGFR less than 60 mL/min per 1.73 m2 (18).

Overweight was defined as BMI 23-24 kg/m² and obesity as 25 kg/m² or more because these cutoffs have been recommended as more reasonable thresholds to define overweight and obesity for Asians (19).Based on the American Diabetes Association criteria of 2014, diabetes was defined as a previous diagnosis by healthcare professionals, FPG ≥ 7.0 mmol/L, or HbA1c ≥ 6.5%. Insulin resistance was estimated by the homeostasis model assessment index of insulin resistance (HOMA-IR): (fasting insulin [milli international units per liter]) * (FPG [millimoles per liter])/(22.5) (20). Central obesity was defined as waist circumference 90 cm or more in men and 80 cm or more in women (21). Hypertension was identified by a systolic BP more than or equal to 140 mm Hg, a diastolic BP more than or equal to 90 mm Hg, or a self-reported previous diagnosis of hypertension by a physician. According to the modified National Cholesterol Education Program-Adult Treatment Panel III, dyslipidemia was defined as total cholesterol more than or equal to 6.22 mmol/L, triglycerides more than or equal to 2.26 mmol/L, LDL-C more than or equal to 4.14 mmol/L or HDL-C less than 1.04 mmol/L, or a self-reported previous diagnosis of hyperlipidemia by physicians (16). ALD and other liver diseases including secondary causes of hepatic fat accumulation such as significant alcohol consumption, use of a steatogenic medication, etc.

### Statistical analysis

SPSS Statistics, version 24 (IBM Corp) was used to perform the statistical analyses. All analyses were 2-sided. P <0.05 was considered statistically significance. Marginal effect indicated 0.05 < P <0.1. Continuous variables were expressed as the mean ± SD, and categorical variables were described as a percentage (%). Characteristics of the study sample were compared by the independent sample t-test or ANOVA for continuous variables, and Pearson Chi square test for categorical variables. The associations of MAFLD and NAFLD with CVD, CKD, and their risk factors were assessed via linear and logistic regression. Age and smoking were adjusted.

Participants with liver steatosis, normal BMI, no type 2 diabetes, and one metabolic risk abnormalities are potential patients of MAFLD because of missing hsCRP data, so sensitivity analysis was performed to include them as MAFLD patients. Their cardiovascular and renal burden was analyzed by regression analysis.

## Results

### Clinical characteristics of participants with and without MAFLD

The clinical characteristics of the participants with and without MAFLD were demonstrated in **Table 1**. The obesity status (BMI, waist circumference, overweight/obesity, central obesity), glucose metabolism (FPG, insulin, HbA1c, HOMA-IR, diabetes), lipid metabolism (TG, TC, HDL-C, LDL-C, dyslipidemia) and blood pressure (systolic blood pressure, hypertension) were worse in MAFLD group (P<0.001 for both male and female). The ALT, AST, and hepatic steatosis by ultrasound were also higher in MAFLD group (P<0.001 for both male and female). Naturally, the cardiovascular burden (Framingham risk score, previous CVD) were heavier in MAFLD group (P<0.001 for both male and female). However, eGFR (P<0.001 for both male and female) was lower in MAFLD group, but the prevalence of CKD was comparable between MAFLD and non-MAFLD group (P=0.299 for male, and P=0.256 for female).

**Table 1:** Clinical characteristics of participants with and without MAFLD.

	Non-MAFLD	MAFLD	P value
**Male**			
N	2296	2639	
age, years	56 ± 14	55 ± 13	< 0.001
current smoking, %	47.14	46.99	0.931
BMI, kg/m²	23.05 ± 2.89	26.49 ± 3.08	< 0.001
Waist Circumference, cm	79.92 ± 8.56	88.92 ± 8.42	< 0.001
FPG, mmol/L	5.49 ± 1.28	5.96 ± 1.74	< 0.001
insulin, pmol/L	31.63 ± 33.01	50.45 ± 67.85	< 0.001
HbA1c, %	5.52 ± 0.86	5.84 ± 1.17	< 0.001
HOMA-IR	1.16 ± 1.88	2.03 ± 4.29	< 0.001
TG, mmol/L	1.38 ± 1.01	2.31 ± 2.27	< 0.001
TC, mmol/L	4.98 ± 0.94	5.29 ± 1.2	< 0.001
HDL-C, mmol/L	1.41 ± 0.32	1.25 ± 0.29	< 0.001
LDL-C, mmol/L	2.96 ± 0.72	3.27 ± 0.78	< 0.001
ALT, U/L	21.73 ± 15.48	28.47 ± 18.94	< 0.001
AST, U/L	26.41 ± 13.19	28.04 ± 18.21	< 0.001
Systolic blood pressure, mmHg	131.39 ± 21.14	136.58 ± 19.87	< 0.001
overweight/obesity, %	47.2	91.1	< 0.001
Central obesity, %	12.28	45.55	< 0.001
Diabetes, %	10.18	22.4	< 0.001
Hypertension, %	44.09	59.88	< 0.001
Dyslipidemia, %	29.18	59.67	< 0.001
Hepatic steatosis by ultrasound, %	6.62	100	< 0.001
ALD and other liver diseases, %	0.52	17.58	< 0.001
Framingham risk score	0.17 ± 0.15	0.22 ± 0.18	< 0.001
Previous CVD, %	8.49	10.25	0.042
eGFR, mL/min/1.73 m²	87.69 ± 14.84	85.79 ± 14.52	< 0.001
CKD, %	3.53	4.13	0.299
**Female**			
N	3996	3252	
age, years	51 ± 14	58 ± 11	< 0.001
current smoking, %	2.33	1.99	0.366
BMI, kg/m²	22.67 ± 3	26.41 ± 3.39	< 0.001
Waist Circumference, cm	73.75 ± 8.39	83.91 ± 8.64	< 0.001
FPG, mmol/L	5.34 ± 1	5.9 ± 1.63	< 0.001
insulin, pmol/L	36.31 ± 24.3	55.01 ± 55.35	< 0.001
HbA1c, %	5.32 ± 0.69	5.81 ± 1.07	< 0.001
HOMA-IR	1.27 ± 1.11	2.17 ± 3.09	< 0.001
TG, mmol/L	1.24 ± 0.82	1.87 ± 1.39	< 0.001
TC, mmol/L	5.07 ± 1.06	5.44 ± 1.16	< 0.001
HDL-C, mmol/L	1.53 ± 0.31	1.37 ± 0.3	< 0.001
LDL-C, mmol/L	3.02 ± 0.79	3.39 ± 0.83	< 0.001
ALT, U/L	16.89 ± 11.87	22.09 ± 14.83	< 0.001
AST, U/L	23.13 ± 10.1	25.76 ± 16.04	< 0.001
Systolic blood pressure, mmHg	125.55 ± 21.41	137.29 ± 20.83	< 0.001
overweight/obesity, %	41.33	88.14	< 0.001
Central obesity, %	22.67	68.57	< 0.001
Diabetes, %	6.78	20.61	< 0.001
Hypertension, %	31.72	58.31	< 0.001
Dyslipidemia, %	25.11	51.62	< 0.001
Hepatic steatosis by ultrasound, %	2.43	100	< 0.001
ALD and other liver diseases, %	0.08	4.31	< 0.001
Framingham risk score	0.03 ± 0.03	0.05 ± 0.06	< 0.001
Previous CVD, %	6.65	12.82	< 0.001
eGFR, mL/min/1.73 m²	89.97 ± 16.46	85.26 ± 14.81	< 0.001
CKD, %	3.83	4.37	0.256

### Clinical characteristics of participants with/without MAFLD and NAFLD

To better demonstrate the difference of clinical characteristics between participants diagnosed with NAFLD and MAFLD, the Non-NAFLD MAFLD and Non-MAFLD NAFLD participants were compared in **Table 2**. Generally, compared with Non-NAFLD MAFLD participants, the Non-MAFLD NAFLD participants have better obesity status (BMI, waist circumference, overweight/obesity, central obesity), glucose metabolism (FPG, insulin, HbA1c, HOMA-IR, diabetes), lipid metabolism (TG, TC, HDL-C, LDL-C, dyslipidemia), blood pressure (systolic blood pressure, hypertension), liver function and steatosis (ALT, AST, hepatic steatosis), cardiovascular burden (Framingham risk score, previous CVD) and eGFR (P<0.05 for both male and female). However, the CKD prevalence was higher in Non-NAFLD MAFLD participants than Non-MAFLD NAFLD ones only in female (P=0.004).

**Table 2:** Clinical characteristics of participants with and without MAFLD and NAFLD. .

	Control	Non-NAFLD MAFLD	Non-MAFLD NAFLD	NAFLD MAFLD
**Male**				
N	2156	464	140	2175
age, years	57 ± 13	**56 ± 10#**	47 ± 17*#	54 ± 13
current smoking, %	47.86	56.58*#	36.43*	44.9
BMI, kg/m²	23.18 ± 2.92	26.76 ± 2.81*#	21.18 ± 1.39*#	26.44 ± 3.13
Waist Circumference, cm	80.27 ± 8.61	91.37 ± 7.84*#	74.5 ± 5.35*#	88.39 ± 8.45
FPG, mmol/L	5.52 ± 1.31	5.95 ± 1.66*	5.02 ± 0.53*#	5.96 ± 1.75
insulin, pmol/L	32.01 ± 33.91	45.73 ± 37.53*#	26.03 ± 12.15*#	51.43 ± 72.54
HbA1c, %	5.55 ± 0.87	6.04 ± 1.14*#	5.09 ± 0.45*#	5.8 ± 1.17
HOMA-IR	1.18 ± 1.93	1.78 ± 1.93*	**0.83 ± 0.39#**	2.08 ± 4.63
TG, mmol/L	1.39 ± 1.03	2.29 ± 2.03*	**1.12 ± 0.56#**	2.31 ± 2.32
TC, mmol/L	5 ± 0.94	5.57 ± 1.38*#	4.72 ± 0.9*#	5.23 ± 1.15
HDL-C, mmol/L	1.41 ± 0.32	1.3 ± 0.31*#	1.49 ± 0.31*#	1.24 ± 0.29
LDL-C, mmol/L	2.98 ± 0.72	3.53 ± 0.79*#	2.79 ± 0.64*#	3.21 ± 0.77
ALT, U/L	21.8 ± 15.67	27.37 ± 16.33*	**20.51 ± 12.19#**	28.71 ± 19.45
AST, U/L	26.56 ± 13.43	30.72 ± 29.61*	**24.1 ± 8.21#**	27.47 ± 14.62
Systolic blood pressure, mmHg	132.14 ± 21.22	140.2 ± 19.9*#	119.6 ± 15.81*#	135.8 ± 19.78
overweight/obesity, %	50.31	92.64*	0*#	90.77
Central obesity, %	13.08	59.05*#	0*#	42.67
Diabetes, %	10.84	25*	0*#	21.84
Hypertension, %	45.83	70.47*#	16.91*#	57.6
Dyslipidemia, %	30.28	63.36*	12.14*#	58.89
Hepatic steatosis by ultrasound, %	0.56	100*	100*	100
ALD and other liver diseases, %	0.56	100*#	**0**	0
Framingham risk score	0.18 ± 0.15	0.24 ± 0.18*	0.1 ± 0.09*#	0.22 ± 0.18
Previous CVD, %	8.81	**9.94**	3.65*#	10.32
eGFR, mL/min/1.73 m²	87.46 ± 14.93	**88.31 ± 13.19#**	91.24 ± 12.88*#	85.25 ± 14.73
CKD, %	3.66	2.16#	1.43	4.55
**Female**				
N	3902	140	94	3112
age, years	52 ± 14	59 ± 9*	45 ± 11*#	57 ± 11
current smoking, %	2.33	6.47*#	2.17	1.78
BMI, kg/m²	22.7 ± 3.01	26.5 ± 3.11*	21.21 ± 1.42*#	26.4 ± 3.4
Waist Circumference, cm	73.85 ± 8.43	85.03 ± 8.04*	69.87 ± 5.11*#	83.86 ± 8.66
FPG, mmol/L	5.35 ± 1	5.84 ± 1.62*	**4.97 ± 0.38#**	5.9 ± 1.64
insulin, pmol/L	36.41 ± 24.48	53.23 ± 46.63*	32.54 ± 14.72*#	55.09 ± 55.7
HbA1c, %	5.33 ± 0.69	5.97 ± 1.02*#	5.07 ± 0.34*#	5.8 ± 1.07
HOMA-IR	1.28 ± 1.12	2.19 ± 3.05*	**1.03 ± 0.49#**	2.17 ± 3.09
TG, mmol/L	1.25 ± 0.83	1.82 ± 1.65*	0.97 ± 0.3*#	1.87 ± 1.38
TC, mmol/L	5.08 ± 1.06	5.52 ± 1.03*	**4.87 ± 0.94#**	5.44 ± 1.17
HDL-C, mmol/L	1.53 ± 0.31	1.37 ± 0.29*	**1.53 ± 0.29#**	1.37 ± 0.3
LDL-C, mmol/L	3.02 ± 0.79	3.57 ± 0.75*#	**2.96 ± 0.72#**	3.39 ± 0.83
ALT, U/L	16.93 ± 11.97	21.08 ± 13.6*	**15.21 ± 6.67#**	22.14 ± 14.88
AST, U/L	23.17 ± 10.18	**25.36 ± 10.72**	**21.35 ± 5.36#**	25.78 ± 16.24
Systolic blood pressure, mmHg	125.87 ± 21.5	140.31 ± 22.13*	111.98 ± 10.56*#	137.15 ± 20.76
overweight/obesity, %	42.33	92.14*	0*#	87.96
Central obesity, %	23.22	77.14*#	0*#	68.19
Diabetes, %	6.94	21.43*	0*#	20.57
Hypertension, %	32.47	61.43*	0*#	58.17
Dyslipidemia, %	25.33	50*	15.96*#	51.69
Hepatic steatosis by ultrasound, %	0.08	100*	100*	100
ALD and other liver diseases, %	0.08	100*#	**0**	0
Framingham risk score	0.03 ± 0.03	0.06 ± 0.06*	0.01 ± 0.01*#	0.05 ± 0.05
Previous CVD, %	6.73	17.86*	**3.33#**	12.59
eGFR, mL/min/1.73 m²	89.86 ± 16.48	83.06 ± 14.77*	94.16 ± 14.83*#	85.36 ± 14.81
CKD, %	3.92	7.86*	**0#**	4.21

*indicates P < 0.05 for the difference between control and Non-NAFLD MAFLD/Non-MAFLD NAFLD groups.

# indicates P < 0.05 for the difference between MAFLD NAFLD and Non-NAFLD MAFLD/Non-MAFLD NAFLD groups.

Bold text indicates P < 0.05 for the difference between Non-NAFLD MAFLD and Non-MAFLD NAFLD groups.

Moreover, the Non-MAFLD NAFLD participants even have better metabolic status (BMI, waist circumference, insulin, HbA1c, systolic blood pressure, overweight/obesity, Central obesity, Diabetes, Hypertension, Dyslipidemia), CVD outcome (Framingham risk score) and CKD outcome (eGFR) than Non-NAFLD Non-MAFLD participants in both male and female (P<0.05). Meanwhile, The Non-NAFLD MAFLD participants have worse metabolic status (HbA1c, LDL-C, central obesity) than NAFLD MAFLD participants in both sexes (P<0.05).

### Burden of MAFLD and NAFLD on hypertension, dyslipidemia, diabetes, central obesity, and overweight/obesity

The different burden of MAFLD and NAFLD on CVD and CKD risk factors were shown in **Table 3**. The odds ratios of MAFLD and hypertension, dyslipidemia, diabetes, overweight/obesity, and central obesity were higher than those of NAFLD in both male and female after adjusting age and current smoking. The beta coefficients of MAFLD and systolic blood pressure, LDL-C, fasting glucose, HbA1c, BMI, and waist circumference were higher than those of NAFLD in both male and female.

**Table 3:** Risk of hypertension, dyslipidemia, diabetes, central obesity, overweight and obesity in MAFLD and NAFLD patients.

	Male		Female	
	MAFLD	NAFLD	MAFLD	NAFLD
	OR (95%CI)	OR (95%CI)	OR (95%CI)	OR (95%CI)
**Hypertension**	2.3 (2.03,2.61)	1.48 (1.3,1.67)	2.35 (2.11,2.62)	2.08 (1.87,2.32)
**Dyslipidemia**	3.65 (3.23,4.12)	2.31 (2.05,2.59)	2.59 (2.33,2.88)	2.43 (2.19,2.7)
**Diabetes**	2.82 (2.38,3.34)	1.87 (1.6,2.19)	2.93 (2.5,3.43)	2.63 (2.26,3.07)
**Overweight & Obesity**	11.58 (9.86,13.61)	4.15 (3.6,4.79)	9.47 (8.34,10.75)	6.63 (5.89,7.48)
**Central obesity**	6.63 (5.7,7.73)	2.71 (2.38,3.08)	6.53 (5.85,7.3)	5.25 (4.71,5.85)
	**B (95% CI)**	**B (95% CI)**	**B (95% CI)**	**B (95% CI)**
**Systolic blood pressure**	6.03 (4.93,7.14)	2.61 (1.49,3.72)	7.47 (6.54,8.41)	6.23 (5.3,7.17)
**LDL cholesterol**	0.31 (0.27,0.35)	0.12 (0.08,0.17)	0.26 (0.23,0.3)	0.24 (0.2,0.27)
**Fasting glucose**	0.5 (0.42,0.59)	0.37 (0.28,0.45)	0.41 (0.35,0.47)	0.37 (0.31,0.43)
**glycosylated hemoglobin**	0.36 (0.3,0.41)	0.19 (0.13,0.24)	0.36 (0.32,0.4)	0.32 (0.28,0.36)
**Body mass index**	3.44 (3.27,3.61)	2.28 (2.1,2.47)	3.52 (3.37,3.68)	3.18 (3.02,3.33)
**Waist circumference**	9.18 (8.7,9.66)	5.57 (5.05,6.1)	8.64 (8.25,9.03)	7.76 (7.37,8.16)

Data are expressed as OR/B (95% CI). All analyses were adjusted for age and current smoking.

### Burden of MAFLD and NAFLD on CVD and CKD

**Figure 2:**
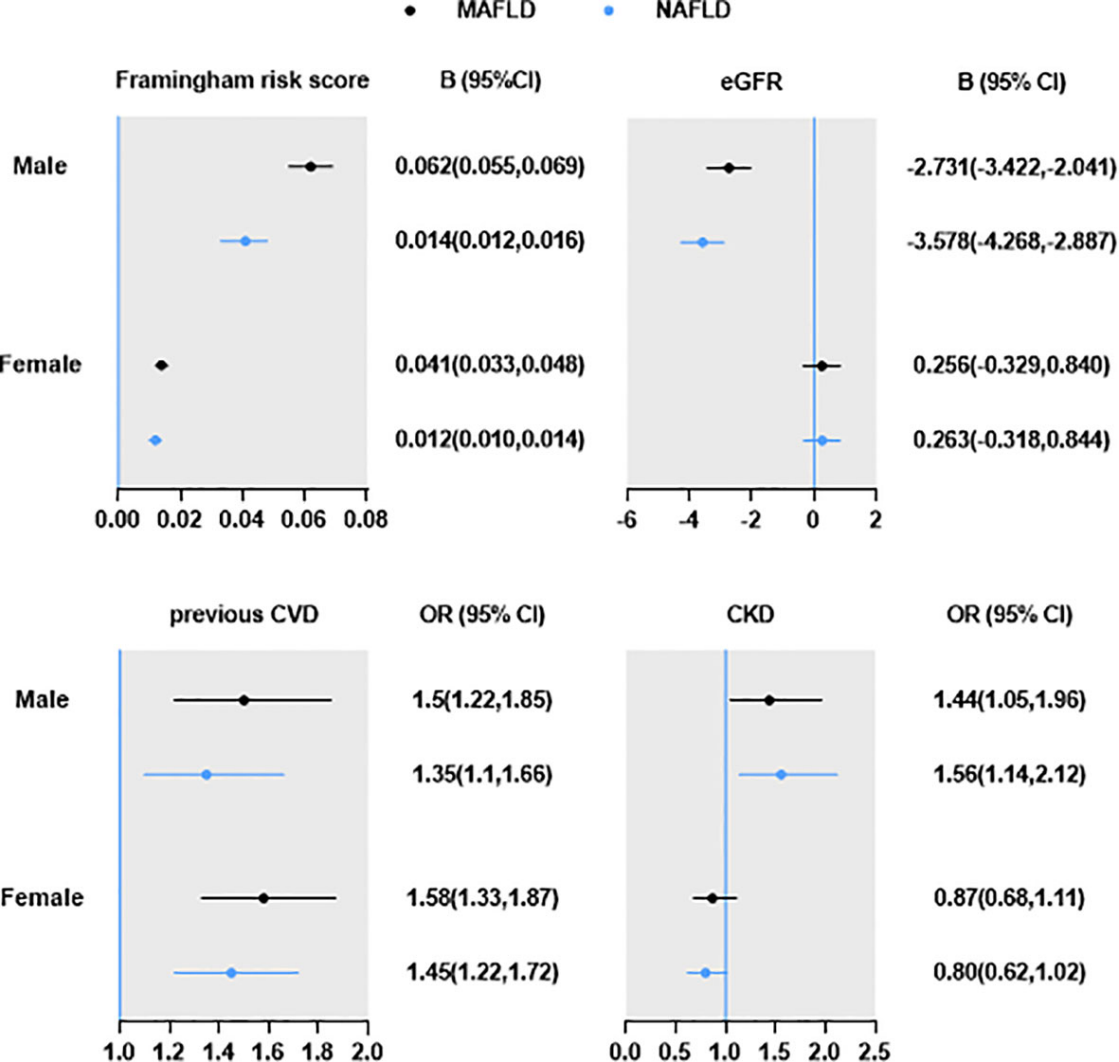
Burden of MAFLD and NAFLD on CVD and CKD. Data are expressed as OR/B (95% CI). All analyses were adjusted for age and current smoking.

The burden of MAFLD and NAFLD on CVD and CKD were shown in **Figure 2**. After adjusting age and smoking, male and female with MAFLD have a similar or higher beta coefficients of Framingham risk score and odds ratios of previous CVD compared with those with NAFLD. However, the odds ratios of CKD was higher in male with NAFLD. In female, CKD was only marginally associated with NAFLD, but not MAFLD.

### Sensitivity analysis

Because hsCRP was not examined in the study population, it was not included in the evidence of metabolic dysregulation. Therefore, sensitivity analysis was performed to analysis the cardiovascular and renal burden of participants with liver steatosis, normal BMI, no type 2 diabetes, and one metabolic risk abnormalities are potential patients of MAFLD (assuming their hsCRP level >2mg/L, n=196). The new MAFLD population (n=6087) still have high beta coefficients Framingham risk score [beta coefficients (95%CI): male 0.06 (0.05,0.07), female 0.01 (0.01,0.02)], and odds ratios of previous CVD [odds ratios (95%CI): male 1.422 (1.15,1.76), female 1.56 (1.32,1.86)]. The renal burden of MAFLD in male remained significant [eGFR -2.73 (-3.42,2.04). CKD 1.42 (1.15,1.76)]. In female, MAFLD was associated with CKD [1.56 (1.32,1.86)], but not eGFR [0.20 (-0.38,0.78)].

## Discussion

In this study, we found that the metabolic status, liver function and hepatic steatosis by ultrasound, and CVD burden were worse in MAFLD group, but the prevalence of CKD was comparable between MAFLD and non-MAFLD groups. Compared with Non-NAFLD MAFLD participants, the Non-MAFLD NAFLD participants have better metabolic status, liver function and steatosis, cardiovascular burden and eGFR. Furthermore, the risk of hypertension, dyslipidemia, diabetes, overweight/obesity, and central obesity of MAFLD patients were higher than those of NAFLD after adjusting age and current smoking. Patients with MAFLD have a similar or higher risk of Framingham risk score and previous CVD compared with those with NAFLD. However, the risk of CKD was higher in male with NAFLD. In female, CKD was only marginally associated with NAFLD, but not MAFLD. Therefore, the new diagnosis criteria of MAFLD could better represent the metabolic and CVD burden of fatty liver than NAFLD.

The association between CVD and NAFLD has been widely recognized, and with the new diagnosis criteria of MAFLD, the association is remained and tend to be stronger. Several pathophysiological mechanisms contribute to CVD including systemic inflammation, hepatic insulin resistance, lipid abnormalities, etc (22). With prospective data from a 5‐year follow‐up study in individuals with type 2 diabetes, NAFLD was significantly associated with an increased risk of CVD independent of diabetes duration, HbA1c, and metabolic risk factors (23). In this study, we found that the risk of CVD and 10-year CVD risk were higher in individuals with MAFLD than with NAFLD both in male and female. Since more independent risk factors of CVD were included in the MAFLD diagnosis (liver steatosis with T2DM, insulin resistance, lipid abnormalities, etc.) than NAFLD (liver steatosis), the MAFLD diagnosis could reasonably lead to higher risk of CVD.

The relationship between NAFLD and CKD is convincing (24). NAFLD diagnosed with liver ultrasound was independently associated with the incidence of CKD (25). There was a higher prevalence of CKD in people with NASH and with advanced fibrosis compared with simple steatosis and non-advanced fibrosis, respectively (26). The common pathophysiology of NAFLD and CKD could influence the its development, including insulin resistance, dyslipidemia, mediators that promote the progression of inflammation, coagulation, oxidative stress and fibration (11).

In a previous study based on Asian population, evidence was found that compared to NAFLD, MAFLD patients were more related to high-risk diseases (27), assuming MAFLD patients should have had higher prevalence of CKD than NAFLD patients. However, in this study, we found that the risk of participants with MAFLD to impair renal clearance and develop CKD were not higher compared with those without MAFLD. The potential mechanism of this phenomenon might have great clinical significance in the application of the new diagnosis criteria. The major difference of non-MAFLD NAFLD and non-NAFLD MAFLD groups were the metabolic status and the pathogenesis that lead to secondary liver steatosis (e.g. excess alcohol consumption, viral hepatitis, specific medications). Most of the individuals with liver steatosis but excluded from NAFLD diagnosis were due to excess alcohol consumption (479 in 604 participants, 79.3%). The role of alcohol consumption in the development of CKD remained in debate.

Alcohol consumption was found to be positively or inversely associated with CKD in various studies. A cohort study including 1883 individuals with CKD during a follow-up of 5555 person-years found that, compared with non-drinking, regular and occasional binge drinking were associated with the risk of CKD progression (28). While another study revealed inverse associations between alcohol consumption and CKD (29). Similarly, the prevalence of stage 3 CKD was lower in drinkers than non-drinkers, and the reverse association between alcohol consumption and stage 3 CKD was found in men (30). Based on the above studies, the difference of CKD risk between MAFLD and NAFLD could be a result of alcohol consumption. Alcohol consumption could be taken into consideration when assess CKD risk in MAFLD patients.

Furthermore, taking a deeper look into the underlie mechanism of the effects of ethanol on the kidneys, it can be found that ethanol is involved in several pathological processes of CKD development. Ethanol can directly cause inflammatory injury in the kidney, independent of liver damage, cause oxidative stress-related damage in the kidneys, influence the interaction of RAS overactivity, hypertension, NO, and prostaglandin E2 deficiency and cause an adverse effect on the kidney morphological structure and renal function (31). Moreover, long-term ethyl alcohol consumption can activate both intrinsic and extrinsic pathways of apoptosis in the kidneys, but aggravates renal fibrosis, which may be related to epithelial mesenchymal transdifferentiation and fibrosis induced by ethanol (32, 33). However, low-concentration ethanol also improves the antioxidant capacity of the renal cells (31). Alcohol consumption could. Therefore, considering that the role of alcohol in CKD is complex, the risk of patients with both MAFLD and excess alcohol consumption should be carefully evaluated.

There are several limitations in this study. First, as a cross-sectional study, causality could not be concluded. Second, hsCRP has great clinical significance in MAFLD inflammation progression, and was included in the diagnosis criteria, but it was not measured in the study population. Therefore, in sensitivity analysis, potential patients of MAFLD (with liver steatosis, normal BMI, no type 2 diabetes, and one metabolic risk abnormalities) were defined as MAFLD and analyzed for their cardiovascular and renal burden of MAFLD, and the burdens were not changed.

In conclusion, the risk of hypertension, dyslipidemia, diabetes, overweight/obesity, and central obesity of MAFLD patients were higher than those of NAFLD. Patients with MAFLD have a similar or higher risk of future and previous CVD compared with those with NAFLD, but the risk of CKD was higher in male with NAFLD. The heterogeneity of MAFLD makes it urgent for endocrinologist and hepatologist to further investigate the characterization of MAFLD, to develop appropriate stratification, and to precisely define subtypes of the disease, so that the prevention and early intervention of intra-hepatic and extra-hepatic adverse outcomes of MAFLD could be ensured and improve their health status and quality of life.

## Data availability statement

The raw data supporting the conclusions of this article will be made available by the authors, without undue reservation.

## Ethics statement

The study protocol was reviewed and approved by the Ethics Committee of Shanghai Ninth People’s Hospital, Shanghai JiaoTong University School of Medicine (approval number 2013 (86)). All procedures followed were in accordance with the ethical standards of the responsible committee on human experimentation (institutional and national) and with the Helsinki Declaration. Written informed consent was obtained from all participants in the study.

## Author contributions

YL, LY and NW designed the study. YW, YY, HZ, CC, HW, YC, FX and SY conducted the research. YW, YY, HZ and NW analyzed the data and wrote the manuscript. The finalmanuscript was read and approved by all authors.

## Funding

This study was supported by National Natural Science Foundation of China (91857117); Science and Technology Commission of Shanghai Municipality (20ZR1432500, 18410722300, 19140902400, 20015800400); the Major Science and Technology Innovation Program of Shanghai Municipal Education Commission (2018YFC1705103); Shanghai Municipal Human Resources and Social Security Bureau (2020074); Clinical Research Plan of SHDC (SHDC2020CR4006). The funders played no role in the design or conduct of the study, collection, management, analysis, or interpretation of data or in the preparation, review, or approval of the article.

## Acknowledgments

The authors thank all team members and participants in the SPECT-China study.

## Conflict of interest

The authors declare that the research was conducted in the absence of any commercial or financial relationships that could be construed as a potential conflict of interest.

## Publisher’s note

All claims expressed in this article are solely those of the authors and do not necessarily represent those of their affiliated organizations, or those of the publisher, the editors and the reviewers. Any product that may be evaluated in this article, or claim that may be made by its manufacturer, is not guaranteed or endorsed by the publisher.
